# Adenosine and Prostaglandin E_2_ Production by Human Inducible Regulatory T Cells in Health and Disease

**DOI:** 10.3389/fimmu.2013.00212

**Published:** 2013-07-25

**Authors:** Theresa L. Whiteside, Edwin K. Jackson

**Affiliations:** ^1^Department of Pathology, University of Pittsburgh Cancer Institute, Pittsburgh, PA, USA; ^2^Department of Pharmacology, University of Pittsburgh School of Medicine, Pittsburgh, PA, USA

**Keywords:** cancer inducible regulatory T cells, natural regulatory T cells, tumor microenvironment

## Abstract

Regulatory T cells (Treg) play a key role in maintaining the balance of immune responses in human health and in disease. Treg come in many flavors and can utilize a variety of mechanisms to modulate immune responses. In cancer, inducible (i) or adaptive Treg expand, accumulate in tissues and peripheral blood of patients, and represent a functionally prominent component of CD4+ T lymphocytes. Phenotypically and functionally, iTreg are distinct from natural (n) Treg. A subset of iTreg expressing ectonucleotidases CD39 and CD73 is able to hydrolyze ATP to 5′-AMP and adenosine (ADO) and thus mediate suppression of those immune cells which express ADO receptors. iTreg can also produce prostaglandin E_2_ (PGE_2_). The mechanisms responsible for iTreg-mediated suppression involve binding of ADO and PGE_2_ produced by iTreg to their respective receptors expressed on T effector cells (Teff), leading to the up-regulation of adenylate cyclase and cAMP activities in Teff and to their functional inhibition. The potential for regulating these mechanisms by the use of pharmacologic inhibitors to relieve iTreg-mediated suppression in cancer suggests the development of therapeutic strategies targeting the ADO and PGE_2_ pathways.

## Introduction

Regulatory T cells (Treg), a small subset of CD4+ T lymphocytes (∼5%) in the peripheral blood, maintain immune responses in balance and ensure that potentially dangerous excessive immune reactivity is prevented. Treg specialize in suppressing responses of other immune cells (1, 2). Recent attention to Treg has been fueled by findings that implicate these cells in several human diseases including cancer, chronic infections, and autoimmune syndromes (3–5). While in health, thymus-derived natural (n) Treg are responsible for peripheral tolerance and immune homeostasis, their imbalance in disease appears to contribute to pathological processes and thus, has been of great interest and importance. Accumulating data suggest that human Treg comprise several distinct subsets of regulatory cells (6), introducing a possibility of a complex regulatory network in which Treg participate but which is orchestrated by factors that remain largely unknown. The question of what or who regulates Treg has been often asked, remains unanswered, and stimulates investigation into Treg interactions with other immune and non-immune cells and into molecular mechanisms underpinning these interactions. In cancer, for example, Treg are thought to be involved in tumor escape from the host immune system (4, 7). And although it is clear that Treg accumulate in tumor tissues and the peripheral circulation of cancer patients (7, 8), the role these Treg play in tumor progression or regression has not been clear, and associations between the Treg frequency and disease outcome remain a subject of a considerable dispute (9). This is a clinically relevant dispute, because if Treg promote cancer progression by interfering with anti-tumor immunity, they need to be muzzled. But if Treg down-regulate inflammatory responses that may favor tumor progression, then their therapeutic removal is contraindicated. At the heart of the controversy is a notion that not all Treg are the same, that their diversity may be environmentally regulated and that they represent a finely regulated system of check and balances which could be therapeutically manipulated to benefit the host. In this paper, we will present evidence in support of this view of human Treg, addressing their characteristics and functions in patients with cancer as well as potential pharmacologic strategies for Treg regulation.

## Problems with the Detection and Isolation of Human Treg

Much of what is currently known about Treg comes from studies in the mouse. Human Treg are difficult to study for several reasons. They are a minor subset of CD4+ T cells for which no definite identifying marker exists. The FOXP3 transcription factor, which has been widely used to study murine Treg, is not a reliable marker of human Treg, because it can be expressed by activated CD4+ T effector cells (Teff) or tissue cells (10–12) and may not be expressed in some activated Treg (13). Also, FOXP3 is an intracellular factor and thus cannot be used for Treg isolation. A high level of CD25 expression on the Treg cell surface is useful in separating Treg from CD25^neg^ cells but is neither specific for Treg (activated CD4+ T cells are IL-2R+) nor particularly helpful in flow cytometry, where a distinction between “high” and “intermediate” IL-2R expression becomes arbitrary. Similarly, *in situ* studies of Treg based on expression of FOXP3 in paraffin sections or the CD4+CD25+ cell frequency in cryosections may not be entirely reliable, and concerns exist that variable results for the Treg frequency in various human tumors, for example, may be the result of methodological differences rather than actual differences in cell counts. Negative selection of Treg based on low or absent expression of CD127 (IL-7 receptor) is often used in mice for Treg enrichment (14), but in man, it may not yield sufficient numbers of high purity Treg. Other surface molecules known to be expressed on Treg, including CTLA-4, GITR, PD-1, ICOS, and chemokine receptors, CCR4, CCR6, and CCR7, endow these cells with special functional characteristics (15–19) but are not specific to Treg and therefore cannot be used for Treg enrichment or isolation. Thus, there is a need for a Treg-specific surface marker that would allow for the selective isolation of human Treg in numbers necessary for their functional characterization.

The discovery of ectonucleotidases, CD39 and CD73, on the surface of murine Treg (20, 21) has focused attention on these enzymes as potential markers of Treg in man. Their expression on the cell surface and enzymatic activity responsible for hydrolysis of exogenous (e) ATP to 5′-AMP and adenosine (ADO) were attractive features which promised to facilitate studies of human Treg. However, a more extensive evaluation of the distribution of these ectoenzymes on human lymphocytes indicated that while CD39 expression was largely restricted to CD4+CD25^high^FOXP3+ T cells, that of CD73 was not, as small subsets of CD4+ as well as CD8+ T cells were found to be CD73+ but CD39^neg^ (22). Furthermore, only<1% of human Treg in the circulation of normal donors co-expressed both enzymes on the cell surface as seen by flow cytometry (23). In Western blots of sorted CD4+CD25^high^ Treg, weak expression of CD73 together with strong CD39 expression was detectable, suggesting an intracellular localization of CD73. Relative levels of mRNA specific for these enzymes in the isolated subsets of CD4+CD39+ and CD4+CD73+CD39^neg^ T cells also indicated the presence of low levels of mRNA for CD73 in the former and of mRNA for CD39 in the latter (23). Confocal microscopy of permeabilized CD4+CD39+ cells showed CD39 evenly distributed on the cell surface, and only rare intracytoplasmic granular inclusions of CD73. In CD4+CD73+CD39^neg^ cells, granular distribution of CD73 in the cytosol was prominent, and surface staining of CD4+CD25+ T cells for CD73 indicated a cap-like staining pattern, suggestive of rapid stripping of this molecule from the cell surface (23). This is in agreement with the reported sensitivity of CD73, a dimer of two identical 70 KDa subunits anchored to the plasma membrane via a C-terminal serine residue linked to glycophosphatidyl inositol (GPI), to proteolytic cleavage (24). Thus, the absence of CD73 from the surface of human CD4+CD39+ Treg may be explained by its rapid turnover and removal from the cell surface associated with a concomitant decrease in the number of intracytoplasmic granules in these cells (23). The rapid utilization and removal of CD73 from the surface of human Treg accompanied by the persistent and intense CD39 expression on their surface suggests that these cells are always prepared to hydrolyze eATP to 5′-AMP, which may either accumulate, signal via A_1_R expressed on Treg or Teff (25) or be further hydrolyzed by CD73 to ADO, depending on the availability of this enzyme on the cell surface. This suggests a carefully orchestrated production of ADO by Treg and the existence of regulatory cellular mechanisms responsible for maintaining collaboration between the two ectoenzymes. Because CD39 is a stable, specific, and enzymatically active-surface marker of human Treg, whose expression levels correlate with that of FOXP3 (26), it has been increasingly often used as a phenotypic/functional marker appropriate for isolation and enrichment of human Treg from human blood and tissues (27).

## Human Natural (n) Treg vs. Inducible (i) Treg

Human Treg can be broadly divided into (a) thymus-derived natural (n) Treg which are present in the periphery of normal donors and regulate tolerance to self and (b) adaptive or inducible (i) Treg, which arise in response to cognate antigens presented by antigen-presenting cells (APC) and expand in the microenvironment enriched in the cytokines promoting Treg proliferation (7). Natural (n) Treg constitutively express FOXP3 and the activation marker CD25 (CD4+CD25^high^FOXP3+), originate in the thymus by high-affinity interaction of the T-cell receptor (TcR) with antigens expressed in the thymic stroma (28), and suppress proliferation of Teff in a contact-dependent, cytokine-independent manner (8). We believe that Treg which accumulate in the peripheral blood and tissues of patients with cancer largely represent iTreg. These cells are induced in the periphery or at tissue/tumor sites from the naïve CD4+CD25(−) T cells in the presence of IL-10 or TGF-β (29) and exert suppression by the production of soluble suppressor factors. Their suppressor functions may not be associated with high levels of FOXP3 expression. These cells are functionally heterogenous and may be broadly subdivided into “activated” (CD25+FOXP3+) cells which express CD45RO (i.e., have a memory phenotype) and “resting” CD45RA+ cells which do not mediate suppression (6, 30–32). Different subsets of iTreg have been recently identified that appear to be phenotypically and functionally distinct from other Treg. These include CD4+CD39+ Treg involved in the ADO pathway (see above), IL-35 producing iTreg (iTreg35) which do not express FOXP3 and are independent of IL-10 or TGF-β (33) and the iTreg subsets that express select chemokine receptors and mediate suppression of only those Teff lineages that utilize the corresponding chemokines (6). The emerging view of iTreg suggests that these Treg develop and function in response to unique microenvironmental stimuli and represent a “tailor made” system of brakes and balances needed to modulate different types of Th responses during inflammation. Thus, various existing immunosuppressive pathways, such as, e.g., the ADO pathway, seem to be able to recruit or induce iTreg which undergo functional specialization resulting in the appearance of Treg able to regulate this pathway. It remains unclear how evolving inflammatory responses regulate this selective Treg specialization process or which environmental stimuli provoke its progression.

## Characteristics of *In vitro* Generated Human iTreg

To be able to learn more about the precise mechanisms responsible for the generation, phenotype, and functions of human iTreg, we developed an *in vitro* assay system for their expansion. Human CD4+CD25^neg^ T cells and autologous immature dendritic cells (iDC) were co-incubated with irradiated tumor cells and a cytokine mix containing IL-2, IL-10, and IL-15 (20 IU/mL of each) for 10 days at 37 °C (34). The cells that outgrew in these cultures gradually acquired phenotypic and functional characteristics consistent with those of iTreg or Tr1 cells as initially described in the literature (29). By day 10, most of proliferating T cells were CD3+CD4+CD25+IL-2Rβ+IL-2Rγ+FOXP3+IL-10+TGF-β+IL-4(−), and they strongly suppressed proliferation of autologous responder T cells (34). Using this well-defined model system for the Tr1 generation, we investigated CD39 and CD73 ectonucleotidase expression on these cells and their potential contribution to ADO-mediated suppression of Teff functions. By flow cytometry and Western blots, most Tr1 cells co-expressed CD39 and CD73 and efficiently hydrolyzed exogenous ATP to ADO as shown in ATP consumption assays and by mass spectrometry for ADO (22). Further, in the presence of ARL67156, a selective CD39 antagonist, or αβ-methylene ADP, an inhibitor of CD73, Tr1-mediated suppression of proliferation of autologous CSFE-labeled CD4+CD25(−) responder T cells was significantly blocked, restoring the ability of these cells to proliferate or produce cytokines (22). In aggregate, these *in vitro* data suggested that human Tr1 co-expressing CD39 and CD73 could produce immunosuppressive ADO and could exert strong suppressive effects on Teff functions via engaging A_2A_R, as ZM241865, a selective A_2A_R antagonist, reversed suppression mediated by Tr1 (22). However, it remained unclear whether *in vivo* generated Tr1, presumably the major subset of iTreg in cancer patients, also co-expressed these ectoenzymes. In the peripheral circulation of normal donors, we and others consistently show expression of CD39 on the surface of nearly all nTreg and of CD73 on only a small (less than 1%) subset of these cells (23). This finding created a need for an explanation of how CD39+ nTreg produce ADO and mediate suppression in the absence CD73 on their surface. One potential explanation may be that CD73 is present in the cytoplasm of Treg and that its expression on the cell surface might be transient and dependent on the state of cellular activation. Recently, we have confirmed the presence of numerous CD73+ granules in the cytoplasm of circulating T and B lymphocytes by confocal microscopy (23). We also showed that CD73 readily aggregates, forming caps on the cells surface of nTreg, which contrasts with its prominent and apparently less transient expression in the *in vitro* generated iTreg (23). Further, we reported that CD4+ T cells expressing CD39 or CD73 were present in tumor tissues (HNSCC), and that at least some CD4+CD25+ Treg infiltrating these tumors co-expressed the two markers *in situ* (13). Another possibility, yet to be investigated, is that CD4+CD39+ Treg producing 5′-AMP could signal via A_1_R and directly modulate activities of Teff, because 5′-AMP has been shown to be an A_1_R agonist independent on ectonucleotidases and capable of binding to A_1_R with an affinity equal to or better than ADO (25). Also, we have recently reported that a CD4+CD73+CD39(−) subset of T cells, most CD19+ B cells which are CD39+CD73+ and CD39+CD73+ exosomes isolated from the plasma of NC or cancer patients are all good ADO producers in the presence of exogenous ATP (23). As T cells, B cells, and exosomes are ubiquitous components in the blood, body fluids, and tissues, we have suggested that they could deliver membrane-tethered CD73 to enable CD39+ Treg to produce ADO. In fact, co-culture experiments in which CD4+CD39+ Treg were co-incubated with any of the CD73+ lymphocyte subsets or exosomes carrying CD39 and CD73 confirmed the validity of this cooperative mechanism for ADO production from eATP (23). Exosomes carrying biologically active CD39 and CD73 were isolated from the plasma of normal controls and were enriched in the cancer patients’ body fluids, suggesting that exosomes derived from CD73+ tumor cells in body fluids of cancer patients may be an especially rich source of CD73 enabling CD4+CD39+ human Treg to produce ADO. The role of exosomes in the regulation of the ADO pathway by delivering their cargo to Treg is a new and intriguing aspect of immune suppression in the tumor microenvironment, although exosomes obtained from the plasma of normal donors also carry CD39 and CD73 ectonucleotidases.

## Accumulations of iTreg in Malignancy

While nTreg develop in the thymus through a series of steps requiring specific signals and transcription factors (2) *de novo* differentiation from naïve CD4+CD25(−) T cells at mucosal sites or in the tumor microenvironment also significantly contributes to the pool of peripheral Treg. It has been well documented that the frequency of circulating Treg is generally increased in patients with various solid tumors or hematologic malignancies (9, 35). Also, Treg percentages are substantially elevated among tumor infiltrating lymphocytes (TIL). Furthermore, suppressive functions are usually much more evident in cancer-associated Treg relative to those present in normal peripheral blood (8). The origin of Treg accumulating in the cancer microenvironments is not clear. Increased recruitment of Treg to tumor microenvironments, which is in part mediated by tumor-derived chemokines and chemokine receptors expressed by Treg (36), could result from enhanced proliferation of Treg in response to tumor antigens or to Treg differentiation and their prolonged survival induced by tumor-derived factors. This potentially diverse origin of tumor-associated Treg might be reflected in their heterogeneity and suggests that the Treg phenotype and functions might be regulated by the local environment. Thus, iTreg induced locally and expanded by soluble factors secreted by tumors are likely to represent the majority of suppressor lymphocytes present in the tumor milieu.

While iTreg which expand and accumulate at tumor sites are expected to suppress anti-tumor immunity and thus favor tumor progression, they also have the ability to block inflammatory responses and thereby reduce or inhibit tumor growth. At present, it is unclear whether blocking of tumor-induced inflammation by iTreg is beneficial to the host. In some human solid tumors, notably colorectal cancer and breast cancer, iTreg frequency and activity *in situ* are reported to predict better outcome (37, 38). In other solid tumors, Treg accumulations seem to be associated with poor prognosis (39, 40). It remains to be determined whether Treg accumulate and influence tumor progression or whether their frequency simply serves as a prognostic marker with no functional impact on cancer progression and outcome. In colorectal cancer, IL-17 expressing Treg subset exists within RORγt-expressing Treg, and these cells expand in late stages of the disease (41). These IL-RORγt-expressing Treg have the potential to produce IL-17, are not suppressive but rather pro-inflammatory and are pathogenic, as they promote disease progression in man and the development of polyposis in mice (42). Expression of RORγt by Treg has been associated with Treg plasticity, a loss of suppressive properties, and conversion to Th-17 (41). Thus, cancer-associated inflammation, at least in colon carcinoma, appears to be controlled by the balance between suppressive Treg and pro-inflammatory RORγt-expressing Treg, although the origin and regulatory elements driving the differentiation of these cell subsets are not yet clear. Thus, there is a need for a better understanding of the mechanisms responsible for Treg accumulations in cancer-induced inflammation. This is a critically important question for future cancer therapies aiming at the elimination of Treg as one means of improving clinical responses.

## Tumor Microenvironment and Human iTreg

Tumor-derived soluble factors, such as VEGF, SDF-1, IL-10, and TGF-β, have been acknowledged to be responsible for expansion of iTreg in tumor-bearing hosts (43, 44). Recently, the number and variety of these factors have been increased to include tumor-derived exosomes which carry death receptor ligands contributing to apoptosis of activated CD8+ Teff (45) as well as a number of other cytokines, chemokines and enzymes able to directly induce expansion of Treg (46, 47). In addition, these factors induce accumulation of immature DC which, in turn, promotes the expansion of Treg, thereby contributing to inhibition of anti-tumor immune responses (48). An enzyme, indoleamine 2,3-dioxygenase (IDO), produced by DC is one of the most potent inducers of Treg differentiation in the tumor milieu (49). The IDO activity results in tryptophan depletion, leading to activation of the GCN2 kinase, and to Treg expansion (50). The ligation of CTLA-4, which is highly expressed on Treg, also leads to enhanced IDO production and favors Treg expansion (51). In addition, the transcription factor, STAT3, as well as the immunosuppressive cytokine, TGF-β, are abundant in the tumor microenvironment and can also contribute to maintaining elevated IDO expression in DC or tumor cells.

In the tumor microenvironment, accumulating CD4+CD39+ iTreg expand upon induction by TA, DC products, and selected cytokines and up-regulate CD73, acquiring the capability to utilize ADO for mediating suppression of other immune cells. In addition to the ADO pathway, another suppressive pathway is known to operate in the microenvironment of many human solid tumors which commonly overexpress cyclooxygenase-2 (COX-2). PGE_2_ is a major product of COX-2 activity, and it too is a powerful immunosuppressive factor often implicated in human tumor progression and poor outcome (52). We reported that *in vitro* generated Tr1 were effective producers of PGE_2_ (53).

## PGE_2_ and iTreg

PGE_2_ mediates immune suppression via EP2 receptors (EP2R), which are G_s_ protein-coupled receptors expressed on the surface of immune cells. Similar to signals processed by ADO A_2A_ receptors (A_2A_R), PGE_2_ signaling leads to an increase in intracellular levels and activation of 3′5′-cAMP in responder cells, with a concomitant decrease in cell proliferation and suppression of cytokine production as well as other immune cell functions (54, 55). PGE_2_ also induces expansion of Tr1 cells and modulates their activity, thus contributing to creating and sustaining a tolerogenic environment (56). We showed that Tr1 proliferation as well as IL-10 and TGF-β production responsible for their suppressor functions were dependent on COX-2 expression in tumor cells (56). When COX-2 expression was inhibited in tumor cells, using siRNA specific for the COX-2 gene or diclofenac, a generic COX inhibitor, Tr1 outgrowth, and suppressor functions were inhibited. Further, tumor cells which overexpressed COX-2 induced a significantly greater number of Tr1 than COX-2(−) tumor cells. Also, Tr1 generated in co-cultures with COX-2+ tumor cells were significantly more suppressive, hydrolyzed more exogenous ATP, and produced higher levels of ADO and PGE_2_ than Tr1 induced by COX-2(−) tumors (56). Tr1 induced by COX-2+ tumor cells were themselves COX-2+ and were able to produce and secrete PGE_2_. These COX-2+ Tr1 co-expressed CD39 and CD73, and in addition to PGE_2_, they also produced ADO (53). Suppressor functions of these Tr1 were blocked in the presence of ectonucleotidase antagonists and also in the presence of indomethacin, confirming that ADO and PGE_2_ contributed to Tr1-mediated immunosuppression (53).

## ADO and PGE_2_ Collaborate in Mediating Suppression in the Tumor Environment

**Figure 1:**
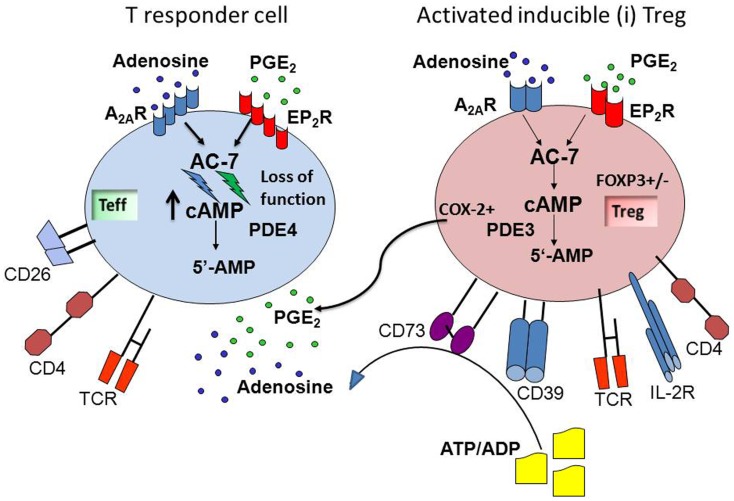
**ADO and PGE_2_ collaborate in mediating suppression in the tumor microenvironment**. Inducible (i)Treg are activated in the tumor microenvironment, co-express CD39 and CD73, and produce ADO via hydrolysis of exogenous ATP/ADP. These Treg also up-regulate COX-2 expression and produce PGE_2_. These two factors, ADO and PGE_2_, are abundant in the tumor microenvironment, which is strongly immunosuppressive. The G-protein-coupled ADO and PGE_2_ receptors on responder T cells receive and process the cognate signals that activate adenylate cyclase-7 (AC-7) and lead to an increase in intracellular levels and activation of 3′,5′-cAMP. This results in suppression of cellular functions in responder T cells. The cooperation between ADO and PGE_2_ is mediated at the level of the AC-7, which together with cellular phosphodiesterase (PDE4) is responsible for regulating 3′,5′-cAMP levels in cells. The ADO and PGE_2_-mediated cooperative inhibition of T effector functions via up-regulation of 3′,5′-cAMP levels represents one of the mechanisms utilized by iTreg for inducing immune suppression.

Since many human solid tumors and Tr1 generated in the presence of these tumors produce ADO and PGE_2_, the tumor microenvironment tends to be immunosuppressive. The G-protein-coupled ADO and PGE_2_ receptors on responder lymphocytes mediate signaling via 3′,5′-cAMP, and the two factors can cooperate in suppressing functions of immune cells (see Figure 1). By adding AH6809, an EP2R antagonist to co-cultures of Tr1 and Teff, we showed that PGE_2_ binds to EP2R on lymphocytes (53). Antagonists of EP1, EP3, or EP4 receptors had no effect on Teff proliferation in these co-cultures. Also, studies with ZM241385, an antagonist of A_2A_R, showed that suppression of Teff proliferation by Tr1-derived ADO was prevented in the presence of this inhibitor, confirming the utilization of A_2A_R on Teff by ADO. In these co-culture experiments, ADO and PGE_2_ appeared to be equally involved in suppression of Teff proliferation by iTreg, as antagonists of EP2 and of A_2_ receptors equally reversed iTreg-mediated suppression (53). As indicated in Figure 1, both ADO and PGE_2_ down-modulate Teff functions by controlling 3′5′-cAMP levels in these cells, presumably by engaging the adenylate cyclase-7 (Ac-7), an Ac isoform present in lymphoid cells (57), as also suggested by our preliminary data (Whiteside and Jackson). This enzyme appears to be a point of convergence for EP2R and A_2A_R, and it contributes to the regulation of 3′,5′cAMP levels in responder cells. Downstream from Ac-7, the protein kinase type I (PKA type I) in effector T cells is also involved in mediating suppressor activity of ADO and PGE_2_ (22). We have shown that Rp-8-Br-cAMPS, an agent which blocks binding of 3′5′-cAMP to the regulatory subunit of PKA type I, significantly inhibited iTreg-mediated suppression of Teff proliferation (53). This observation suggests that blocking of PKA type I activity in Teff could protect them from suppression delivered by ADO- and PGE_2_-producing iTreg.

To determine whether ADO and PGE_2_ play a role in the suppressive activity of Treg *in vivo*, we measured the frequency of circulating CD39+ and COX-2+ Treg by flow cytometry in cohorts of patients with HNSCC at various disease stages (8, 53, Schuler et al., in revision). The frequency of CD39+ or COX-2+ Treg was increased in these patients’ blood (relative to that in NC), and it correlated with disease progression (8, 23, 58, Schuler et al., in revision). Suppressor function of these Treg was also significantly increased (8). Further, co-expression of CD39 and COX-2 in iTreg present among TIL in HNSCC tissues was observed by immunohistochemistry (22). Our data support the conclusion that iTreg present in the blood and tumor tissues of patients with cancer co-express CD39 and COX-2 and have the capability to produce ADO and PGE2. While CD39 and COX-2 were co-expressed in circulating CD4+ T cells of HNSCC patients, IL-10 and TGF-β were expressed by a non-overlapping, distinct subset of CD4+ T cells. These data suggested that Treg producing ADO and PGE_2_ may be distinct from the Treg subset expressing IL-10 and TGF-β. Further, in HNSCC patients successfully treated with chemoradiotherapy (CRT) and evaluated during post-therapy clinical remission, only CD39+, but not IL-10+ or TGF-β+ Treg, were found to be expanded and to accumulate. This CD4+CD39+ subset of Treg was shown to be resistant to CRT, persisted in the patients’ circulation for months after CRT and mediated high levels of immune suppression (Schuler et al., in revision). This observation suggests that CD39+ iTreg might be of special clinical significance *in vivo*, because their suppressive activity could facilitate the disease recurrence. In HNSCC, the disease recurrence within 2–3 years of successful oncological therapies occurs in a large proportion (50–60%) of patients. Therefore, the possibility that CD39+ iTreg contribute to early recurrence by inhibiting anti-tumor immune responses is being further investigated at our institution in a prospective non-therapeutic clinical trial.

If ADO and PGE_2_ produced by activated iTreg synergize in mediating suppression of conventional T cell functions, the result of such synergy is powerful immune suppression of immune cells. Because human tumors are often COX-2+ and are rich in extracellular ATP due to cell death, opportunities exist for ATP-mediated up-regulation of ectonucleotidase activities and COX-2 expression in iTreg generated and accumulating in the tumor microenvironment. In fact, up-regulation in expression and activity of these enzymes is known to occur during inflammation, which is a frequent component of the tumor development (54, 59). The cooperation between the ADO and PGE_2_ pathways, which is regulated at the 3′,5′-cAMP level, is an example of a powerful suppressor mechanism which, by down-regulating anti-tumor immune responses, contributes to tumor progression, and tumor escape from immune control.

## ADO- and PGE_2_-Producing iTreg as Pharmacologic Targets in Cancer

A number of clinically applicable pharmacologic interventions exist for direct interference with the production of ADO and/or PGE_2_ or with binding to their cognate receptors on immune cells. Pharmacologic interventions have been used to block undesirable suppressive effects of these factors in diseases other than cancer (58, 60, 61). The inhibitors of the PGE_2_ pathway (e.g., celecoxib or indomethacin, diclofenac, ibuprofen) have been previously utilized in cancer therapy (61). However, the application of pharmacologic inhibitors to specifically target iTreg accumulating in cancer is a novel therapeutic strategy. To date, Treg depletion has depended on the delivery to tumor-bearing hosts of low-dose cyclophosphamide, daclizumab (anti-CD25 Ab), denileukin diftitox (ONTAC), or tyrosine kinased inhibitors such as sunitinib (62–64). Largely utilized to diminish suppression and improve endogenous anti-tumor immunity, these agents have transient and inconsistent effects on the Treg frequency and functions. More recent use in the clinic of ipilimumab or anti PD-1/PDL-1 Abs, which target T-cell checkpoints including those operating in Treg, might be more effective in controlling suppression, but their effectiveness is still being evaluated. In patients with cancer, iTreg able to produce ADO and PGE_2_ accumulate in tissues and blood and may be resistant to conventional oncological therapies (Schuler et al., in revision), so that silencing of these cells appears to be advisable. Pharmacologic agents such as inhibitors of ectonucleotidase activity, A_2A_R or EP2R antagonists or inhibitors of PKA type I activity can effectively block suppression mediated by iTreg, as shown in our *in vitro* experiments (22). In addition, rolipram, a phosphodiesterase 4 (PDE4) inhibitor, increased 3′,5′-cAMP levels in Teff thereby increasing their susceptibility to iTreg-mediated suppression (53). Drugs blocking COX-2 activity are in clinical use and can be readily selected for blocking PGE_2_ production by iTreg and thus prevent suppression of Teff. With evidence pointing to A_2A_R and EP2R as the negative signal-mediating receptors in lymphocytes, it might be rational to design the pharmacologic blockade specifically targeting these receptors. Alternatively, the selective blockade in Teff of Ac-7, which is the convergence point for ADO and PGE_2_ signaling, is expected to restore anti-tumor activity in patients with cancer (65, 66). Because the Ac-7 isoform integrates signals generated by both ADO and PGE_2_ pathways and are expressed mainly, perhaps exclusively, in hematopoietic cells (67), it represents a potentially attractive therapeutic target. Inhibition of Ac-7 activity by pharmacologic agents could be confined to lymphocytes, leading to selective down-regulation of 3′,5′-cAMP levels in Teff, up-regulation of Teff functions, silencing of iTreg and relief from ADO- and PGE_2_-mediated suppression. Unfortunately, among the available pharmacologic inhibitors of Ac none is specific for the Ac-7 isoform, and further development is necessary for implementing the simultaneous blockade of ADO and PGE_2_ pathways at the point of their convergence. Nevertheless, this remains an attractive possibility for restoration of immune competence in cancer and represents a novel strategy for “blocking the inhibitors” with pharmacologic agents.

Yet another pharmacologic intervention that could lead to restoration of anti-tumor immunity involves the PDE pathway in Teff. Levels of 3′,5′-cAMP in Teff are partly determined by the activity of PDEs, and its up-regulation with, e.g., propanolol, leading to a decrease in cAMP levels, can be expected to restore Teff functions and decrease Treg-mediated suppression. A recent study in mice, illustrated the *in vivo* effectiveness of a PDE-directed pharmacologic strategy (68). Bushell et al. showed that stimulation of CD4+ T cells by allogeneic DC in the presence of cilostamide, an inhibitor of PDE3, resulted in a significant increase in the number and functions of Treg, which blocked allograft rejection (68). This *in vivo* study confirms that modulation of PDE activity is a promising strategy for controlling functions of Treg.

## Conclusion

Among suppressive mechanisms utilized by Treg in patients with cancer, ADO- and PGE_2_-mediated suppression appears to be especially prominent. These factors are present in the tumor microenvironment not only because many human tumors produce them but also because activated iTreg, the subtype of Treg accumulating in tissues and the peripheral circulation of cancer patients, are also ADO and PGE_2_ producers. iTreg express enzymes involved in ATP hydrolysis and PGE_2_ production, and utilize ADO and PGE_2_ to up-regulate 3′5′-cAMP in Teff suppressing their functions. Pharmacologic interventions designed to selectively target components of the ADO and/or PGE_2_ pathways could not only inhibit the tumor-derived factors but also to silence suppressive functions of Treg and thus restore anti-tumor activity of Teff. A particularly attractive therapeutic strategy for overcoming tumor-induced immune suppression and prevent tumor escape involves a blockade by pharmacologic agents of cooperative interactions between ADO and PGE_2_. Pharmacologic blocking of this cooperation, which is mediated via the Ac-7 isoform present in lymphocytes and responsive to A_2A_ and EP2 receptor signaling, depends on the future development of small molecular weight selective inhibitors of Ac-7 activity. The resulting alterations in cAMP levels in Teff could restore their anti-tumor functions and silence Treg in cancer.

## Conflict of Interest Statement

The authors declare that the research was conducted in the absence of any commercial or financial relationships that could be construed as a potential conflict of interest.
